# A Machine Learning-Based Guide for Repeated Laboratory Testing in Pediatric Emergency Departments

**DOI:** 10.3390/diagnostics15151885

**Published:** 2025-07-28

**Authors:** Adi Shuchami, Teddy Lazebnik, Shai Ashkenazi, Avner Herman Cohen, Yael Reichenberg, Vered Shkalim Zemer

**Affiliations:** 1 Department of Mathematics, Ariel University, Ariel 4070000, Israel; 2 Department of Cancer Biology, Cancer Institute, University College London, London WC1 9BT, UK; 3 Adelson School of Medicine, Ariel University, Ariel 4070000, Israel; 4 Faculty of Medical and Health Sciences, Tel Aviv University, Tel Aviv 6997801, Israel; 5 Clalit Health Services, Dan-Petach Tikva District, Petach Tikva 4900000, Israel

**Keywords:** health economics, quality assurance, pediatric emergency department, laboratory tests, community, children, healthcare, data-driven models

## Abstract

**Background/Objectives**: Laboratory tests conducted in community settings are occasionally repeated within hours of presentation to pediatric emergency departments (PEDs). Reducing unnecessary repetitions can ease child discomfort and alleviate the healthcare burden without compromising the diagnostic process or quality of care. The aim of this study was to develop a decision tree (DT) model to guide physicians in minimizing unnecessary repeat blood tests in PEDs. The minimal decision tree (MDT) algorithm was selected for its interpretability and capacity to generate optimally pruned classification trees. **Methods:** Children aged 3 months to 18 years with community-based complete blood count (CBC), electrolyte (ELE), and C-reactive protein (CRP) measurements obtained between 2016 and 2023 were included. Repeat tests performed in the pediatric emergency department within 12 h were evaluated by comparing paired measurements, with tests considered justified when values transitioned from normal to abnormal ranges or changed by ≥20%. Additionally, sensitivity analyses were conducted for absolute change thresholds of 10% and 30% and for repeat intervals of 6, 18, and 24 h. **Results:** Among 7813 children visits in this study, 6044, 1941, and 2771 underwent repeated CBC, ELE, and CRP tests, respectively. The mean ages of patients undergoing CRP, ELE, and CBC testing were 6.33 ± 5.38, 7.91 ± 5.71, and 5.08 ± 5.28 years, respectively. The majority were of middle socio-economic class, with 66.61–71.24% living in urban areas. Pain was the predominant presented complaint (83.69–85.99%), and in most cases (83.69–85.99%), the examination was conducted by a pediatrician. The DT model was developed and evaluated on training and validation cohorts, and it demonstrated high accuracy in predicting the need for repeat CBC and ELE tests but not CRP. Performance of the DT model significantly exceeded that of the logistic regression model. **Conclusions:** The data-driven guide derived from the DT model provides clinicians with a practical, interpretable tool to minimize unnecessary repeat laboratory testing, thereby enhancing patient care and optimizing healthcare resource utilization.

## 1. Introduction

Repeat laboratory testing is a well-documented and widespread practice in healthcare [1,2], accounting for a substantial proportion of overall laboratory utilization [3,4,5,6]. A hospital-based study reported that 20% to 30% of laboratory tests were repeated within a 24 h period [3]. Repeated testing is intended to reduce diagnostic uncertainty, inform therapeutic decision-making, improve patient care [7,8], and acknowledge patients’ clinical presentations [2].

Despite these intended benefits, studies indicated that early repeat laboratory testing yielded negligible or clinically insignificant differences and exerted no influence on clinical decision-making, rendering such repeat tests unjustified [3,9,10]. These heighten patient discomfort, raise healthcare costs, delay clinical decision-making, and impair the effectiveness of clinical management [11]. As highlighted in several studies, although clinicians strive to deliver cost-effective care, limited transparency around laboratory testing expenses may inadvertently lead to occasional overutilization of diagnostic tests [3]. The clinical and financial inefficiencies associated with redundant testing have been highlighted [6,7,11].

In line with these observations, laboratory tests conducted in community settings are occasionally repeated within hours of referral to pediatric emergency departments (PEDs) [1,10]. The prevalence of these repeat tests and the factors driving their occurrence remain poorly understood [1,12]. Limited research has investigated repeat testing in PEDs and pediatric intensive care units [1,11,13]. Pageler et al. [13] implemented a computerized physician order entry intervention in a pediatric intensive care unit that restricted select laboratory tests to a single daily order and required daily reassessment of their necessity. This strategy reduced test utilization, shortened both pediatric intensive care unit and overall hospital lengths of stay, and had no adverse effects on mortality. These findings underscore the importance of clear guidelines to minimize unnecessary repeat laboratory testing in clinical decision-making.

Given the lack of clinical benefit and potential harm of unnecessary repeats, multiple interventions have been proposed to reduce the overuse of laboratory testing [7]. Proposed strategies have ranged from physician education and audit-and-feedback programs to electronic health record enhancements that display cost data, financial incentive schemes, and administrative policies limiting specific test orders. While these interventions have been effective in some settings, their availability and applicability in pediatric care remain limited [14,15]. In pediatric settings, decisions to repeat laboratory tests require consideration of additional complexities, including heterogeneous socio-demographic factors [16]. Tailored strategies in pediatric emergency departments must balance the potential risks posed by repeat laboratory testing with the necessity of comprehensive clinical evaluation.

To translate these considerations into a practical solution, this study aims to equip pediatricians with a practical, data-driven guide to minimize unnecessary repeat blood tests in PEDs. To achieve this, a DT model was constructed using routinely available clinical data and initial community laboratory results to predict when repeat testing was warranted. The resulting, user-friendly tool is designed to reduce test repetitions that are unlikely to affect decision-making.

This study constitutes the first comprehensive application of machine learning to assess the necessity of repeat blood testing in pediatric patients across both community and emergency department settings. The model provides a precise, data-driven framework to assist clinicians in minimizing unnecessary repeated laboratory tests, thereby enhancing patient care, improving healthcare quality, and optimizing resource utilization.

## 2. Materials and Methods

**Figure 1:**
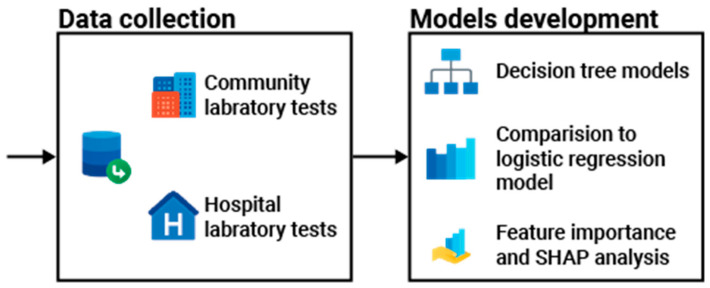
A schematic overview of this study’s methodological framework.

Initially, the study cohort and associated clinical and laboratory data were characterized. Next, a metric to assess the necessity of repeat laboratory tests was established. Finally, the data-driven methodology for developing the clinical decision guide and its statistical validation are presented. Figure 1 demonstrates a schematic view of the methodological flow of this study, including the data collection process and the model development steps.

The study cohort was assembled from Clalit Health Services (CHS), Israel’s largest health maintenance organization, which insures and provides care to approximately 54% of the country’s population. CHS maintains a comprehensive electronic database capturing patient demographics, visits to community clinics, emergency departments, and hospitals, laboratory test results, and medication prescriptions and purchases. Each physician visit includes a diagnosis recorded in accordance with the International Classification of Diseases, Ninth Revision (ICD-9). Data were extracted from CHS’s electronic medical records via the MDClone© analytics platform. The inclusion criteria comprised medical visits of children aged 3 months to 18 years across Israel’s three central administrative districts, between January 1, 2016 and December 31, 2023. Eligible visits included instances in which an initial community laboratory test was repeated in a PED within a 12 h interval.

This study was approved by CHS’s Institutional Review Board for Human Studies (approval number: 0099-21-COM). Signed informed consent was waived, as this retrospective cohort analysis of an existing database did not include any clinical intervention or patient contact.

Six laboratory tests were analyzed and grouped into three categories: complete blood count (CBC), including white blood cell count, hemoglobin, and platelet count; serum electrolytes (ELEs), including sodium and potassium; and C-reactive protein (CRP). Data collected from the electronic medical records included socio-demographic information such as age, sex, and socio-economic status (SES), as well as details of the laboratory tests conducted in the community and later in the PED. The patient’s SES was based on the Israeli Central Bureau of Statistics classification system, which assigns 10 categories of SES [17]. Categorization was based on multiple variables, including demographic characteristics, educational attainment, employment, income levels, and housing conditions. Complete records were available for every patient in the cohort.

With complete demographic and socio-economic data for all patients, this study then defined criteria for necessary repeat testing. In the absence of a standardized definition in the medical literature, two criteria were applied to classify a repetition as justified. The first criterion deemed tests whose results shifted from within the community’s normal reference range to outside the PED’s reference limits as clinically necessary. The second criterion employed an absolute difference of ≥20% between community and PED to denote clinically significant change. The absolute difference threshold was selected because it has been shown to outperform relative percent change in identifying clinically meaningful variations [18]. Sensitivity analyses were performed for absolute difference thresholds of 10% and 30% and inter-test intervals of 6, 18, and 24 h.

Recent advances in machine learning (ML) model applications in medicine [19,20,21,22] highlighted the suitability of decision tree algorithms for clinical decision support. Accordingly, a DT-based model [23], elected for its proven clinical performance and straightforward interpretability [24,25,26], was developed to translate our established criteria into a practical, data-driven guide. The minimal DT (MDT) algorithm [27] was employed to construct the binary DT model. The MDT algorithm was employed because, unlike conventional machine learning methods that iteratively contract decision tree nodes, it performs a global optimization of the entire tree to produce an optimal classification model [28].

To prepare the data for model development, all continuous variables were converted into discrete categories using a binning approach. To improve the generalization of the results, the k-fold (k = 5) cross-validation method was utilized [29], as well as the SAT post-pruning method [30].

The study population was initially divided into training and validation cohorts for model development and evaluation. Statistical tests were then performed to confirm that age and sex distributions were comparable between the two groups. This step was essential to ensure that the validation set reflected the distribution of the training set, a critical requirement for the generalizability of machine learning models in clinical settings [31]. This balance was achieved using the Directed Bee Colony Optimization algorithm [32]. Model performance was evaluated using the receiver operating characteristic area under the curve (ROC-AUC), along with accuracy, sensitivity, specificity, positive predictive value (PPV), negative predictive value (NPV), Cohen’s alpha, and F1 metrics, in accordance with standard practices in medical machine learning [33,34]. For comparison, the widely used logistic regression (LR) model was employed, given its linear structure and interpretability [35].

To explore the decision-making properties of the resulting DT models, two commonly used techniques were applied. First, feature importance was assessed using the standard permutation importance algorithm, which quantifies the average impact of each parameter on the model’s final decision [36]. Second, SHapley Additive exPlanations (SHAP) analysis was applied to further interpret the model by quantifying the contribution of each feature to individual predictions [37,38]. The SHAP values provide a unified measure of feature contribution by attributing the effect of each input variable to a specific model prediction. In feature importance analysis, these values quantify the magnitude and direction of each feature’s influence, with positive values indicating a positive contribution and negative values indicating a negative impact. All analyses were conducted using Python version 3.9.5, with statistical significance defined as *p* < 0.05.

## 3. Results

### 3.1. Study Population

Out of 812,271 pediatric emergency department visits recorded during the study period, 7813 (0.96%) met the inclusion criteria for analysis. Among the included visits, repeated testing was performed in 6044 cases for the complete blood count (CBC), 1941 for electrolytes (ELEs), and 2771 for C-reactive protein (CRP). Some patients underwent more than one type of repeated blood test. The mean ages of patients, stratified by the type of repeated laboratory test, were 5.08 ± 5.28 years for the CBC group, 7.91 ± 5.71 years for the ELE group, and 6.33 ± 5.38 years for the CRP group. The distribution of sexes was balanced, with males comprising 49.43–52.39% across test groups. The majority of patients belonged to the middle SES (66.61–71.24%) and resided in urban areas (66.61–71.24%). Pain was the most frequently reported complaint across all test groups (83.69–85.99%), and the majority of patients were diagnosed by pediatricians (83.69–85.99%). The detailed description of the study populations is presented in Table 1.

### 3.2. Repeated Laboratory Testing

Approximately half of the patients in each blood test group were hospitalized, with rates ranging from 48.38% to 55.56%. Based on the predefined criteria for appropriateness, only 2924 of 6044 (7.32%) CBC tests, 1078 of 1941 (11.85%) ELE tests, and 1383 of 2771 (13.68%) CRP tests were considered appropriate.

### 3.3. Models for Predicting the Necessity of Repeated Laboratory Testing

Table 2 presents the performance metrics of three DT models and three LR models, corresponding to the CBC, ELE, and CRP tests. Model evaluation included accuracy, F1-score, sensitivity, specificity, PPV, NPV, Cohen’s alpha, and ROC-AUC. The F1-score, commonly used in classification tasks, represents the harmonic mean of precision and sensitivity.

Focusing on the DT models, the CBC model demonstrates the highest performance, achieving an accuracy of 78.72% and an F1-score of 72.19%. The similarity between these two metrics suggests that the model generalized well without overfitting. Conversely, the CRP model exhibited lower accuracy and performed only slightly better than random classification, indicating limited predictive value. The ELE model achieved an accuracy of 73.18% and an F1-score of 69.52%. Notably, across all three test categories, the DT model outperformed the LR in terms of accuracy, F1-score, and ROC-AUC. The superior performance of the DT model may be attributed to its ability to capture non-linear relationships. Consequently, we did not assume a global linear association between repeat laboratory testing and individual model parameters, such as patient age. The DT model demonstrated a consistent performance across all evaluated criteria, including the 10%, 20%, and 30% absolute difference thresholds and the out-of-range definition, supporting its robustness. Based on this consistency, the 20% threshold was selected for further analysis, as it demonstrated a balanced performance across all evaluation metrics. Sensitivity analyses for 6, 18, and 24 h intervals between repeated tests are presented in Supplementary Table S1.

Supplementary Figure S1 presents the DT models for the CBC, ELE, and CRP laboratory tests, with each node displaying a decision based on a specific model parameter and threshold. The decision path proceeds to the right if the specified condition is met and to the left if it is not, ultimately reaching a leaf node. Each leaf node indicates the model’s recommendation regarding the necessity of repeating the laboratory test. Notably, the root node (first row) in all DT models was defined by the corresponding laboratory test result obtained in the community setting. In cases involving multiple laboratory tests, such as the CBC model, additional community-based test results appeared immediately after the root node in the decision path. In higher-order decisions nodes (i.e., the upper levels of the tree structure), the socio-demographic characteristics, such as patient age and sex, played a prominent role.

### 3.4. Variables Contributing to the Decision Tree Models

**Figure 2:**
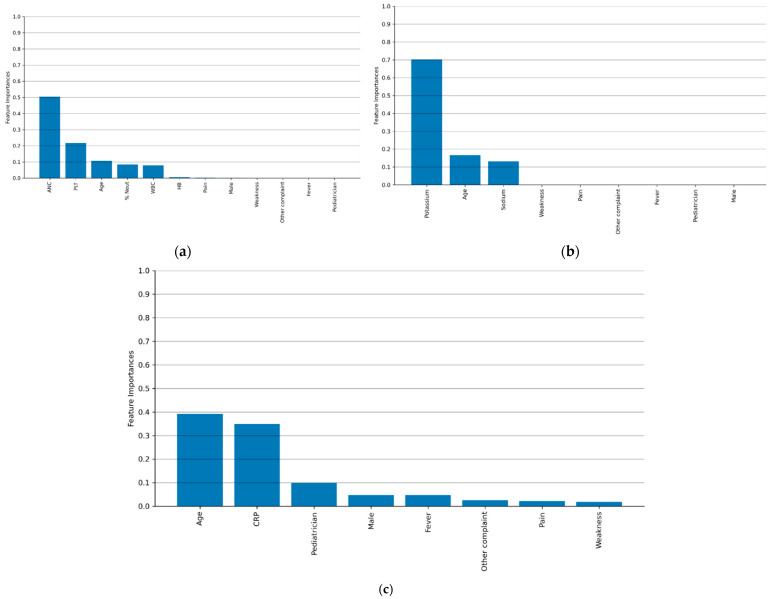
The variables’ relative contributions (feature importance) to the justification for repeat blood testing according to the decision tree models (**a**) CBC, (**b**) ELEs, and (**c**) CRP.

Figure 2a–c illustrate the contributions of various clinical and demographic variables to the decision tree models of the CBC, ELE, and CRP laboratory tests. Feature importance reflects the average contribution of each variable to the model’s predictions, offering an estimate of which features are most informative in determining the necessity of a repeated laboratory test. The laboratory results obtained in the community emerged as the most influential feature, followed by patient age. In contrast, patient sex (represented by the “Male” variable in Figure 2) had minimal to no impact on the model’s predictions, with importance values close to or equal to zero. Furthermore, neither the primary presenting symptom nor the identity of the referring clinician (pediatrician or family physician) demonstrated a measurable predictive value in the models.

**Figure 3:**
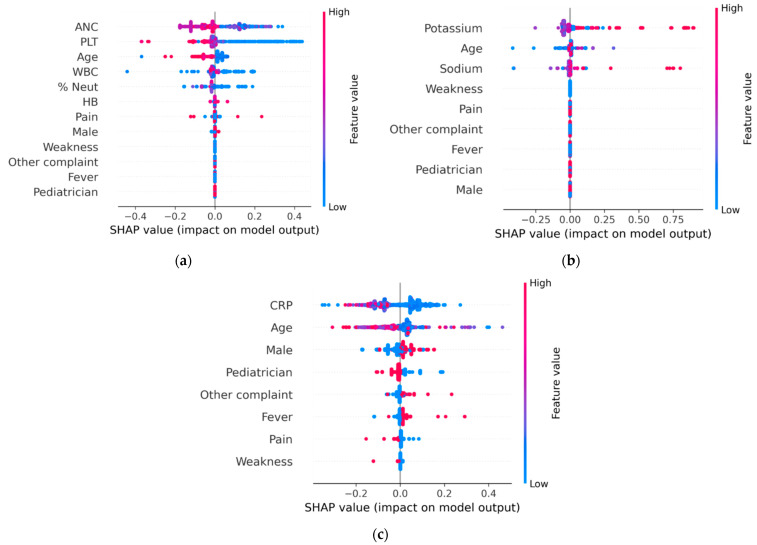
The approximate linear relationship between the decision tree model variables and the predictive need for repeat laboratory testing based on SHAP (Shapley Additive exPlanations) analysis: (**a**) CBC; (**b**) ELEs; (**c**) CRP.

Figure 3 presents the SHAP analysis of the three DT models corresponding to the CBC, ELE, and CRP tests. In the CBC model, the absolute neutrophil count (ANC) measured in the community emerged as a key predictor. Higher ANC values were associated with an increased probability of the model recommending repeat testing, as indicated by the concentration of blue points to the right of the central reference line, along with several overlapping purple points. The second most important feature was the platelet count (PLT), which exhibited a pattern similar to that of ANC. The third was patient age, where younger patients had a slightly higher likelihood of requiring repeat testing. As age increased, the predicted need for repetition decreased, as reflected by the red values appearing to the left of the central line. For the ELE model, serum potassium level was the most influential feature, with elevated values associated with an increased likelihood of repeat testing. The second and third most influential features were patients age and serum sodium level, both of which exhibited less distinct patterns in relation to the predicted need for repeat testing. For the CRP model, lower serum values were associated with a higher likelihood of repeat testing. Patient age was the second most important feature. With respect to sex, male patients were more likely than female patients to undergo repeat CRP testing.

## 4. Discussion

In this study, data-driven guides were developed to assist physicians in determining whether a repeated laboratory test in the PED is warranted when an identical test was recently performed in the community. The guides were based on widely available data, including socio-demographic characteristics and results from the initial community laboratory tests, supporting both their practicality and generalizability.

Initial findings, as presented in Table 2, indicate that the proportion of repeated laboratory tests classified as appropriate is relatively low (7.32–13.68%). This observation is consistent with previous research in pediatric populations, which also reported high rates of seemingly unjustified laboratory test repetition [12]. These findings underscore the challenges clinicians face in accurately assessing the necessity of repeated laboratory tests. They also highlight the potential value of a decision support tool to reduce unnecessary repeat testing, with implications for improving the patient experience, healthcare quality, and cost efficiency [7,8].

When examining individual laboratory tests, the DT-based data-driven guide demonstrated a strong predictive performance for CBC and ELEs, achieving high accuracy in identifying justified repetitions. However, its performance for CFP was limited, showing only a marginal improvement over random classification (Table 2). The limited performance of the CRP model may be attributed to the relatively long half-life of CRP, approximately 48 h, which reduces the likelihood of observing significant changes within a 12 h retesting period. When examining the DT models under a configuration in which false negatives were set to zero, thereby ensuring that no justified repeat tests were incorrectly dismissed, the models achieved a true positive rate higher than that of random guessing, as shown in Figure 2. This configuration indicates that adopting the proposed guides in this manner does not adversely affect clinical outcomes. When compared to the baseline logistic regression model, the DT demonstrated a marginal improvement in predictive accuracy. This difference may be attributed to the non-linear structure of the DT model, in contrast to the linear nature of LR. The result highlights the importance of models that account for multiple interacting variables, rather than relying on a single factor, such as patient age. It is important to note that these tools are intended to support, not replace, clinical judgment; the pediatrician’s assessment and any changes in the patient’s condition remain paramount.

The DT models and clinical guides developed in this study are consistent with established clinical understanding. For example, as demonstrated in Figure 2 and Figure 3, patient age plays an important role in decision-making but remains secondary to clinical status, as reflected by the initial community laboratory results. In support of this observation, Desai et al. conducted an electronic survey among 43 pediatric residents and staff physicians to examine perceptions of laboratory stewardship and testing patterns at a tertiary pediatric hospital [16]. The survey revealed a high level of familiarity with laboratory stewardship, with respondents acknowledging that overuse of laboratory testing is a recognized issue both in general medical practice and within their own institution. The majority of survey respondents indicated that increased cost transparency could help reduce laboratory test overuse. However, they reported limited familiarity with key aspects of test utilization, including test costs, specificity and sensitivity, required blood volumes, and existing clinical guidelines for common laboratory tests. The study showed that 53% of laboratory tests were repeated within 7 days, and only half of these repeat tests produced abnormal results [16]. Similar findings were observed in both pediatric and adult populations [39,40]. In addition, previous studies emphasized the importance of cost transparency in reducing the frequency and cost of blood tests and imaging studies [41,42].

This study has several notable strengths, including the large sample size, the real-world, population-based design, and its practical applicability. The analytical tools developed can be adapted to different threshold criteria, applied to additional laboratory tests, and implemented in various clinical settings to support physicians decision-making. However, several limitations should be acknowledged. First, due to the retrospective design of this study, the proposed analyses and decision guides were simplified, and clinical data such as patients’ medical history were not included. This information could have provided additional insight into the patients’ medical conditions and potentially influenced the assessment of whether repeat blood testing was warranted. Additionally, this study did not assess the actual impact of repeated testing on clinical decision-making or patient outcomes, which should be addressed in future prospective studies. Furthermore, cases in which a justified repeat test was not performed were not examined. Second, in the absence of an established definition in the literature for justified repeat testing, two criteria were applied: a change from a normal to an out-of-normal range result, and an absolute change of ≥20%. Sensitivity analyses were also conducted to support these thresholds. Third, in this analysis, repeat testing was defined as occurring within a 12 h interval. This strict definition captures a specific subset of repeated tests and does not account for those performed over a longer duration, such as during an extended hospital stay or ongoing interactions with the healthcare system. To address this limitation, additional analyses were performed using extended time intervals of 6, 18, and 24 h between repeated tests. The methodology applied in this study can be readily adapted to evaluate other time intervals and alternative definitions for justified repetition thresholds.

## 5. Conclusions

This study presents a novel, data-driven approach for assessing the necessity of repeated blood testing in pediatric emergency settings, grounded in real-world data from both community and hospital settings. The proposed guide supports clinical decision-making and, when used alongside physician judgment, has the potential to reduce unnecessary repeat testing while maintaining high standards of patient care. The tools presented in this study, which are relatively simple and rely on readily available data, can be adapted for use in clinical settings to help reduce unjustified repetition of laboratory testing. Future prospective studies are recommended to evaluate the effectiveness of the proposed model in reducing unnecessary laboratory testing and in supporting clinical decision-making by pediatricians. Demonstrating effectiveness in real-world settings may lead to an improved quality of care, enhanced patient and family experience, and meaningful reduction in healthcare costs and system burden.

## Figures and Tables

**Table 1:** Description of the socio-demographic characteristics of the study population *.

Variable	Repeated Complete Blood Count Test (CBC)	Repeated Electrolyte (Na, K) Tests (ELE)	Repeated CRP (CRP)
Visits, *n*	6044	1941	2771
Hospitalization	2924 (48.38%)	1078 (55.56%)	1383 (49.92%)
Age (years), mean (±SD)	5.08 (5.28)	7.91 (5.71)	6.33 (5.38)
Age group			
3 mo–2 years	2758 (45.63%)	434 (22.37%)	1080 (30.96%)
3–10 years	2001 (33.11%)	752 (38.72%)	1175 (42.40%)
11–18 years	1285 (21.29%)	755 (39.91%)	516 (27.64%)
Sex			
Male	2988 (49.43%)	971 (50.05%)	1452 (52.39%)
Female	2056 (50.57%)	969 (49.95%)	1319 (47.61%)
SES			
Low	1692 (27.99%)	615 (31.68%)	914 (32.98%)
Middle	3125 (51.70%)	903 (46.52%)	1306 (47.13%)
High	1227 (20.31%)	423 (21.80%)	551 (19.89%)
Residence type			
City	4219 (69.80%)	1293 (66.61%)	1974 (71.24%)
Village	1458 (24.12%)	495 (25.50%)	638 (23.02%)
Other	367 (6.98%)	152 (7.89%)	159 (5.74%)

* All values are expressed as *N* (%), unless otherwise stated.

**Table 2:** Performance of decision tree and logistic regression models across thresholds of 10%, 20%, and 30% and out-of-normal-range absolute changes for CBC, CHS, and CRP tests. Metrics: accuracy; F1-score; ROC-AUC; sensitivity; specificity; positive predictive value; negative predictive value; and Cohen’s κ.

Model	Prediction	Condition	Accuracy	F1-Score	ROC-AUC	Sensitivity	Specificity	PPV	NPV	Cohen’s Kappa
Decision tree	CBC	10%	85.81%	78.68%	73.40%	75.48%	89.96%	82.26%	86.97%	68.60%
	ELE	10%	79.78%	75.77%	72.49%	72.57%	82.35%	78.50%	77.01%	56.90%
	CRP	10%	72.46%	70.75%	68.73%	67.55%	75.58%	74.07%	70.36%	44.90%
	CBC	20%	78.72%	72.19%	67.34%	68.99%	85.21%	75.69%	80.46%	55.00%
	ELE	20%	73.18%	69.52%	66.47%	66.32%	77.01%	72.82%	70.81%	45.70%
	CRP	20%	66.48%	64.91%	63.09%	61.71%	70.36%	68.32%	65.04%	33.40%
	CBC	30%	91.70%	84.09%	78.43%	80.89%	93.30%	86.81%	90.76%	79.30%
	ELE	30%	85.24%	81.08%	77.42%	77.88%	87.20%	83.92%	83.18%	68.70%
	CRP	30%	77.44%	75.62%	73.49%	72.42%	80.82%	79.46%	75.62%	56.80%
	CBC	out-of-range	77.15%	70.75%	66.00%	67.55%	84.18%	74.07%	79.16%	52.10%
	ELE	out-of-range	76.84%	72.99%	69.79%	69.79%	80.20%	76.13%	73.80%	51.80%
	CRP	out-of-range	69.80%	68.16%	66.24%	64.96%	73.13%	71.30%	67.92%	39.50%
Logistic regression	CBC	10%	75.57%	73.09%	68.14%	69.89%	78.96%	76.62%	73.71%	49.90%
	ELE	10%	72.87%	70.43%	67.05%	67.23%	76.45%	73.84%	70.93%	44.90%
	CRP	10%	66.00%	65.32%	64.35%	62.12%	70.19%	68.79%	65.55%	34.40%
	CBC	20%	69.33%	67.05%	62.51%	63.85%	72.76%	70.47%	67.57%	38.00%
	ELE	20%	66.85%	64.62%	61.56%	61.42%	70.21%	67.92%	65.05%	33.20%
	CRP	20%	61.48%	59.93%	59.04%	56.73%	65.59%	63.33%	60.19%	23.90%
	CBC	30%	80.76%	78.11%	72.80%	74.91%	84.18%	81.65%	79.16%	60.90%
	ELE	30%	77.85%	75.29%	71.70%	72.09%	81.33%	79.25%	76.44%	54.00%
	CRP	30%	71.62%	70.08%	68.76%	66.88%	74.88%	73.66%	70.76%	44.60%
	CBC	out-of-range	67.94%	65.71%	61.26%	62.51%	71.49%	69.17%	66.27%	35.40%
	ELE	out-of-range	70.19%	67.85%	64.64%	64.65%	73.08%	70.78%	67.92%	38.90%
	CRP	out-of-range	64.55%	62.93%	61.99%	59.73%	68.49%	66.45%	63.36%	29.70%

## Data Availability

All statistical analyses are available upon reasonable request. Because of ethical and privacy issues, patients’ data cannot be shared.

## Supplementary Materials

The following supporting information can be downloaded at https://www.mdpi.com/article/10.3390/diagnostics15151885/s1, Figure S1: DT models for the CBC, ELE, and CRP laboratory tests; Table S1. Model performance (decision tree vs. logistic regression) over the time intervals 6, 12, 18, and 24 h.

## Abbreviations

The following abbreviations are used in this manuscript:

CBC	Complete blood count
CHS	Clalit Health Services
CRP	C-reactive protein
DT	Decision tree
ELEs	Electrolytes
MDT	Minimal DT
PEDs	Pediatric emergency departments
SES	Socio-economic status
