# Supplementary figures and images for: A Machine Learning-Based Guide for Repeated Laboratory Testing in Pediatric Emergency Departments
Source: Diagnostics (Basel). 2025 Jul 28;15(15):1885. doi: 10.3390/diagnostics15151885 (PMC12346183; doi:10.3390/diagnostics15151885)

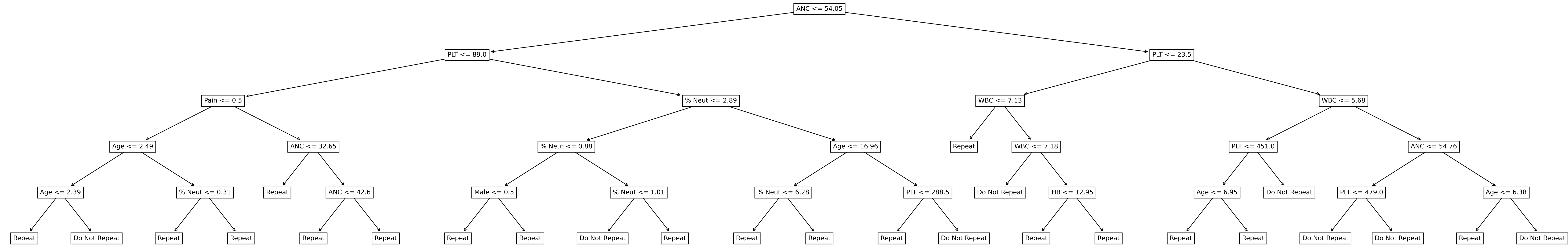

Supplement: Supplementary file 1 [file diagnostics-15-01885-s001.zip › Figure S1- A. CBC.pdf]

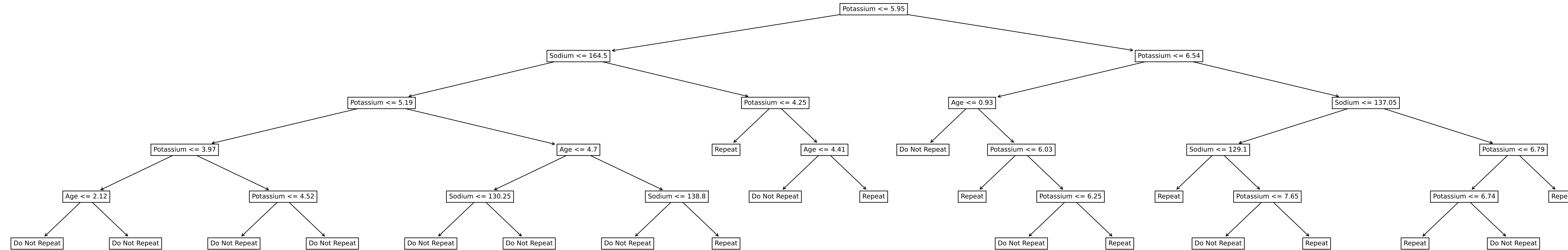

Supplement: Supplementary file 1 [file diagnostics-15-01885-s001.zip › Figure S1- B. ELE.pdf]

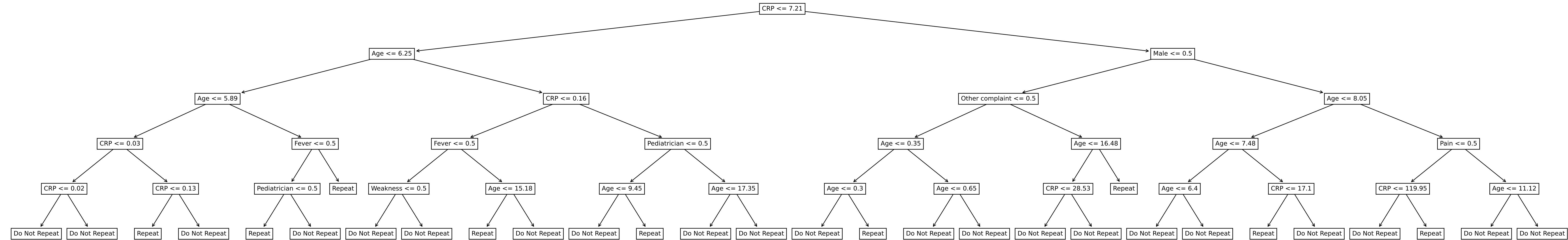

Supplement: Supplementary file 1 [file diagnostics-15-01885-s001.zip › Figure S1- C. CRP.pdf]
